# Anaphylaxis Due to Head Injury

**DOI:** 10.5811/westjem.2015.3.24397

**Published:** 2015-04-09

**Authors:** Heather C. Bruner, David I. Bruner

**Affiliations:** Temecula Valley Hospital, Temecula, California; Naval Medical Center San Diego, San Diego, California

## Abstract

Both anaphylaxis and head injury are often seen in the emergency department, but they are rarely seen in combination. We present a case of a 30-year-old woman who presented with anaphylaxis with urticaria and angioedema following a minor head injury. The patient responded well to intramuscular epinephrine without further complications or airway compromise. Prior case reports have reported angioedema from hereditary angioedema during dental procedures and maxillofacial surgery, but there have not been any cases of first-time angioedema or anaphylaxis due to head injury.

## INTRODUCTION

Anaphylaxis with angioedema is a serious and potentially life-threatening emergency. There are many potential triggers for anaphylaxis. Head trauma has not been previously reported as a trigger for an acute allergic reaction with angioedema. We present a case report of a young woman with mild traumatic head injury who subsequently developed urticaria and angioedema.

## CASE PRESENTATION

A 30-year-old Caucasian female presented to the emergency department (ED) after being hit in the head by a baseball sustaining a laceration to her right lateral forehead. She did not lose consciousness and was driving herself home from a baseball game when she began to develop urticaria, tongue swelling and difficulty breathing. She stopped at a local fire station where her rapidly worsening symptoms were treated with 0.3mg of intramuscular (IM) epinephrine before transport to our ED approximately 30 miles from the fire station.

At the time of her arrival, her urticaria had subsided, but her tongue swelling persisted such that she had difficulty speaking and some discomfort with swallowing but was tolerating her own secretions without difficulty. She denied vomiting or present shortness of breath, but she did initially feel short of breath prior to epinephrine administration. She complained of a headache but no neck pain since the trauma, and she was not confused.

Her physical exam revealed a patient who was appropriately alert and oriented with a Glasgow Coma Score (GCS) of 15 and completely intact neurologic exam. She had a 4cm laceration over the right side of her forehead with minimal bleeding after being bandaged at the local fire station but no other deformity. She was tachycardic with a pulse of 110 beats per minute with a blood pressure of 118/76mmHg. Her respiratory rate was 22 breaths per minute with oxygen saturation of 98% on room air and no active stridor or wheezing noted. Her skin exam revealed several minor urticarial lesions on her anterior neck and trunk but no other lesions.

She reported no history significant past medical or surgical history and denied any allergies or history of allergic reactions. There was no family history of hereditary angioedema, and the patient was not taking any prescribed or over the counter medications. She stated that she had not been stung or bitten, and had ingested no new foods prior to or after being hit by the baseball.

In the ED she received methylprednisolone 125mg intravenous (IV), diphenhydramine 25mg IV, famotidine 20mg IV and one liter of normal saline. A non-contrast computed tomography (CT) of the head was negative for fracture or intracranial hemorrhage. Her forehead laceration was subsequently repaired without difficulty.

After a period of observation in the ED, her tongue swelling had not improved. Because she had persistent tongue swelling and lived more than one hour from the hospital, she was admitted to the hospital for airway observation. She was discharged 18 hours later with improvement in her swelling and no recurrent allergic symptoms.

## DISCUSSION

This presentation represents the first reported case of anaphylaxis occurring as a possible result of blunt head trauma. In attempting to explain the combination of this patient’s presenting symptoms, we considered the possibility that she may have had some unrecognized allergen exposure leading to her anaphylaxis. However, she denied any new exposures either before or after, and she was not taking any medications that could have contributed to a delayed allergic reaction. The alternative explanation remains that the head injury was the proximate cause of her subsequent anaphylaxis, and this combination of these symptoms has not been previously presented in the medical literature.

When patients present to the ED with head trauma, the physician’s primary concern is to evaluate for possible intracranial hemorrhage. In managing these patients one typically tries to avoid potentially increasing the intracranial pressure (ICP) until it is apparent that there is not any intracranial hemorrhage. The conundrum for the management of this patient, in particular by the emergency medical services (EMS) providers, was whether or not a patient with head trauma and a possible intracranial hemorrhage should receive epinephrine to treat her angioedema, which could elevate her mean arterial pressure (MAP) and thus potentially increase her ICP.

The thought process of the emergency medical technician (EMT) was that he should manage her airway first, and he decided to administer an appropriate IM dose of epinephrine that helped to ameliorate, but not resolve, her allergic symptoms. Clearly, the EMT made the correct treatment choice to manage the anaphylaxis first as the patient’s head injury was not severe enough to cause any neurologic deficit or subsequent intracranial hemorrhage. However, had the injury been severe enough to create a space-occupying lesion, the use of epinephrine could certainly contribute to a rise in ICP and potentially worsen the neurologic outcome. Using epinephrine to mitigate an airway and oxygenation emergency would have to be weighed against the possible risk of increasing ICP.

Anaphylaxis due to trauma alone has not been reported in the literature based on our review. Research in rats has suggested that injury to the blood brain barrier in head trauma may possibly contribute to cerebral edema and angioedema because of changes in expression of aquaporin-4 (AQP4), but this has only been studied in rats and little is known about the correlation of angioedema that occurs in rats with traumatic brain injury.^1^

Hereditary Angioedema (HAE) has been reported in several older case reports to be triggered during maxillofacial or dental surgery. There are case reports of acute HAE occurring for the first time during oral surgery for an acute mandibular fracture.^2^,^3^ There are multiple potential triggers for HAE, including many types of physical and psychological trauma.^4^,^5^ However, most of these events have been reported in patients with known HAE. Our patient had no personal or family history of HAE, and her symptoms began with urticaria which is more likely to be present in anaphylactic reaction than HAE which usually is more likely to be swelling that is nonpitting, not raised, and not pruritic as our patient’s symptoms were.^4^,^5^

This patient had a mixed presentation of allergic symptoms that could potentially be attributed to anaphylaxis or HAE. Type 1 allergic reactions are the common cause of anaphylaxis. They are due to IgE mediated mast cell degranulation that causes pruritus, flushing, urticaria, and anaphylaxis within minutes to hours of exposure.^4^,^6^ Our patient could also have had exercise-induced anaphylaxis that can occur from direct or non-immune related mast cell activation up to 4–6 hours after exposure and exercise.^4^ HAE symptoms differ in that urticaria is rare, while there may be nonpitting and nontender perioribital, lip, or tongue swelling.^5^ We contend that our patient had an allergic anaphylactic reaction because she had mucosal and skin findings within hours of “exposure,” but there are no prior reports of trauma triggering anaphylaxis. Trauma, however, is noted in reviews of HAE as a potential trigger, but we could not find any case reports to support this contention. While this could be the case for our patient, her symptoms were more indicative of anaphylaxis, and there was no personal or family history of HAE.

We referred our patient to an allergist for further testing, but she did not go to that appointment. She did follow up with her primary physician within the following week for suture removal and did not report recurrence of her symptoms.

In summary, this was an unusual case of minor head trauma leading to a first-time incidence of anaphylaxis with angioedema. The head injury may have been the triggering mechanism for her allergic reaction, and was managed successfully with one dose of IM epinephrine, diphenhydramine, steroids, and observation. Although HAE symptoms have been reported with head, neck and dental surgery previously, the combination of head injury leading to angioedema has not previously been reported.
